# Monocenter feasibility study of the MRI compatibility of the Evia pacemaker in combination with Safio S pacemaker lead

**DOI:** 10.1186/1532-429X-14-67

**Published:** 2012-09-25

**Authors:** Christian G Wollmann, Erich Steiner, Paul Vock, Bonaventure Ndikung, Harald Mayr

**Affiliations:** 1 Hospital of St. Pölten-Lilienfeld, Department of Cardiology, St. Pölten, Austria; 2 Institute of Diagnostic Imaging, Frühwald, Steiner, Obermayer, St. Pölten, Austria; 3 Karl Landsteiner Society, Institute of Research on Ischemic Heart Diseases and Rhythmology, St. Pölten, Austria; 4 Biotronik SE & Co KG, Berlin, Germany

**Keywords:** Pacemaker, Magnetic resonance, Adverse events, Follow-up, Remote monitoring

## Abstract

**Background:**

The purpose of this study was to evaluate the feasibility of the magnetic resonance (MR) conditional pacemaker (PM) system (Evia SR-T and DR-T with Safio S leads) under MR conditions.

**Methods:**

Patients with standard PM indications and Evia PM were eligible for enrollment in this single center prospective non-randomized pilot study. Patients underwent MR of the brain and lower lumbar spine at 1.5 Tesla. Atrial (RA) und ventricular (RV) lead parameters (sensing, pacing threshold [PTH], pacing impedance) were assessed immediately before (baseline follow-up [FU]) and immediately after MRI (1^st^ FU), after 1 month (2^nd^ FU) and 3 months (3^rd^ FU). The effect of MR on serious adverse device effect (SADE) free-rate, on atrial and ventricular sensing (AS/VS; mV) and atrial (RA) and ventricular (RV) pacing thresholds (PTH; V/0.4 ms) were investigated between baseline and 2^nd^ FU. Continuous variables are expressed as mean ± SD and were compared using paired Student’s t-test. A p < 0.05 was considered significant.

**Results:**

Thirty-one patients were enrolled. One patient had to be excluded because of an enrollment violation. Therefore, data of 30 patients (female 12 [40%], age 73 ± 12 years, dual chamber PM 15 [50%]) were included in this analysis. No MR related SADE occurred. Lead measurements were not statistically different between the baseline FU and the 2^nd^ FU (AS/VS at baseline 3.2 ± 2.1/15.0 ± 6.0, at 2^nd^ FU 3.2 ± 2.1/14.9 ± 6.5; p = ns. RA-PTH/RV-PTH at baseline 0.68 ± 0.18/0.78 ± 0.22, at 2^nd^ FU 0.71 ± 0.24/0.78 ± 0.22; p = ns). The presence of the permanent pacemakers led to MR imaging artifacts on diffusion weighted sequences of the brain, but did not affect other sequences (e.g. FLAIR and T2 weighted spin-echo images).

**Conclusion:**

The use of the MR conditional Evia PM in a MR environment under predefined conditions is feasible. No MR related SADEs nor clinically relevant changes in device functions occurred.

## Background

Magnetic resonance imaging (MRI) has become an important tool for diagnosis and therapy control of many diseases [1]. Patients with cardiac implantable electronic devices such as permanent pacemakers and implantable defibrillators are usually excluded from MRI examinations since the majority of implantable devices have a contraindication for MRI [2,3]. Some larger series with pacemaker patients who underwent MR suggest an acceptable risk-benefit ratio by taking MRI and pacemaker related precautions [4-7]. Potential (adverse) effects of MR on pacemaker function include effects from different sources [2,7]. The static magnetic field may induce mechanical forces on ferromagnetic components, unpredictable magnetic sensor activation, reed-switch closure and changes in electrocardiograms. Potential effects from modulated radio frequency (RF) fields are heating of cardiac tissue adjacent to lead electrodes potentially leading to pacing threshold increases, possible induction of arrhythmias, pacemaker reprogramming or reset, RF interactions with the device (over-and undersensing). Gradient magnetic fields may induce arrhythmias, and voltages on leads causing over- and under-sensing. Combined field effects are alterations of device function because of electro-magnetic interference, mechanical forces (vibration), electrical reset of devices, and damage to pacemaker and/or leads.

As a result of patients’ needs (and their physicians’) different manufacturers recently market released permanent implantable pacemaker systems which are designed with respect to the above mentioned potential adverse effects of MR on permanent pacemakers. The first of these especially designed pacemaker systems has been proven in a prospective, randomized study published showing the safety of a MR conditional dual chamber pacemaker system [8].

By mid of 2010 the MR conditional EVIA pacemaker systems (Biotronik SE & Co KG, Berlin, Germany) became available in Europe, offering the Evia pacemaker family [Evia SR(−T) and DR(−T)] with the first MR conditional single chamber pacemaker system [Evia SR(−T)].

The purpose of our study was to evaluate feasibility of the new MR conditional pacemaker system (Evia SR-T and DR-T with Safio S53/S60 active screw-in leads, Biotronik SE &Co KG, Berlin, Germany) under specific MR conditions. We report the results of the monocenter feasibility study of the MRI compatibility of the Evia pacemaker in combination with the Safio S pacemaker lead.

## Methods

From all patients implanted with permanent pacemakers at the general hospital of St. Pölten-Lilienfeld those patients implanted with Evia SR-T or DR-T PM and Safio S leads at least 6 weeks before intended MR scan were eligible for enrollment in this single center prospective non-randomized pilot study. Pacemaker implantations were indicated according to current guidelines [9,10]. At enrollment pacing thresholds had to be ≤ 2.0 Volts/0.4 ms, pacing impedances between 200 and 1.500 Ohms. The charge state of the battery had to be at least 30%. Patients had to be afebrile. Patients must not have scheduled cardiac surgery within 3 months after enrollment. Patients gave written informed consent at enrollment. The study was approved by the local ethical committee and the Austrian competent authority.

### Evia pacemakers

The Evia pacemakers (single chamber [SR/-T] and dual chamber [DR/-T]) are suited for all standard indications of bradycardia therapy. The contact surfaces of the pacemakers are titanium and epoxy resin. All pacemakers are rate-adaptive and multi-programmable. The Evia pacemaker offers atrial and ventricular capture control, ventricular pace suppression (VpS) algorithm and IEGM transmission via Home Monitoring (SR-T and DR-T only). For this study the Evia pacemakers (SR-T and DR-T) were used together with the Safio S pacemaker leads. These pacemaker systems are conditionally MRI CE-approved. Safety and efficacy of the Evia pacemakers have been approved previously [11].

### Safio s leads

The Safio S is an active-fixation, transvenous, bipolar, endocardial lead designed for permanent atrial or ventricular stimulation and sensing. The lead is insulated with silicone, and has an IS-1 connector. The Safio S is flexible between the tip and ring electrodes and has an isodiametric design with a diameter of 6.6 Fr. The Safio S lead is a follow-up product of the Setrox S lead. The safety and efficacy of the Setrox lead was demonstrated earlier [12].

### Home monitoring

The Evia DR-T and SR-T pacemakers have the ability to transmit and receive data over a distance of several meters using bi-directional long-range telemetry, i.e. without the need of a programming wand. The data is transmitted to a patient device (Cardio Messenger). Via mobile phone technique, the Cardio Messenger forwards the data to the Home Monitoring Service Center. Safety and efficacy of remote monitoring by Home Montoring in pacemaker patients have been demonstrated recently [13].

Within this study, the pacemaker based programmable Home Monitoring parameters were set as follows: automatic transmission time 2:00 am; periodical EGM transmission every 30 days; transmission in case of high atrial frequency and sustained atrial episode (Evia DR-T only); transmission in case of high ventricular frequency; kind of report: trend report.

### MR scans

MR scans were conducted under consideration of previously published recommendations for MR in pacemaker patients [2,4-7]. After given written informed consent patients underwent a non-diagnostic MRI of the brain and the lower lumbar spine at 1.5 Tesla.

Before MR patients were asked for potential contra-indications for MR scan. Tables 1 and 2 show in detail the MR scan protocols for the brain and for the lower lumbar spine, respectively. The brain scan consisted of 9 sequences with a total expected scan time of 14.5 minutes, and a dedicated receiving brain coil was used. Since MR scans were of a non diagnostic intention the last 2 sequences of the brain examination (CE-MRA and Perfusion) were performed without the administration of contrast agent. The lower lumbar spine scan consisted of 6 sequences and was expected to last 14.8 minutes. For the lower lumbar spine scans an incorporated lumbar spine coil was used. Before performing the MR scans pacemakers were programmed to the MR mode (ProMRI feature). Within this feature the pacing mode was programmed according to the requirements of the patients to an asynchronous single chamber (A00 or V00), dual chamber mode (D00) or was deactivated (OFF). In case of programming an asynchronous pacing mode, the lower rate was automatically set to 80 bpm, and the output to 4.8 Volts/1.0 ms. Different pacemaker functions (e.g. capture control, memory functions) were deactivated during active ProMRI mode automatically. Upon programming the pacemaker in the ProMRI mode the Home Monitoring transmission system is automatically rendered inactive (i.e. OFF). Thus no risks of interferences between data transmission and MR system are to be expected.

**Table 1:** MRI sequences for brain scan

	**Sequences for Philips MRI Scanner**								
	**Body Region: Head, Patient Position: Supine, Patient Entry: Head First, Landmark on Eyes**								
	**3 plane localizer**	**Reference Scan**	**AX SE T1**	**AX FSE T2**	**T2 FLAIR**	**Diffusion**	**3D TOF MT**	**CE-MRA**	**Perfusion**
	**Landmark on Eyes**	**Landmark on Eyes**	**Landmark on Eyes**	**Landmark on Eyes**	**Landmark on Eyes**	**Landmark on Eyes**	**Landmark on Eyes**	**Landmark on Eyes**	**Landmark on Eyes**
**Name Anatomy Philips**	Head CNS-Brain	Ref Scans	Head CNS-Brain	Head CNS-Brain	Head CNS-Brain	Head CNS-Function	Head CNS-Angio	Head CNS-CE-Angio	Head CNS-Function
**Sequence Name Philips**	survey	RefScan	T1W_SE	T2W_TSE	T2W_FLAIR	DWI_E	S3DI_MC_HR	3D_DYN_AVM	sPRESTO
**plane**	ax/cor/sag	-	ax	ax	ax	ax	ax	sag	ax
**Scan time**	0:17	0:51	02:39	02:13	03:18	00:36	02:52	00:51	0:50
**stacks**	3	2	1	1	1	1	1	1	1
**slices**	3 each	82	18	18	16	18	100	10	30
**Scan technique**	T1-TFE	SENSE	SE	SE	IR	SE	FFE	FFE	FFE
**Fast imaging mode**	TFE	-	MS	TSE	TSE	EPI	none	none	EPI
**(Turbo/EPI) Factor**	42	-	1	19	23	89	-	-	19
**TE [ms]**	5.2	0.95	15	100	120	104	6.9	1.27	shortest
**TR [ms]**	15	8	483	3607	6000	3034	24	5	22
**TI [ms]**	-	-	-	-	2000	-	-	-	-
**Flip angle [deg]**	20	7	140	90	100	90	20	35	7
**FOV [mm]**	250	-	230	230	230	230	200	260	220
**Scan [%]**	74	82	79	74	68	79.6	57.4	100	55.8
**Scan matrix**	256 x 126	64 x 52	256 x 163	384 x 228	208 x 115	112 x 89	332 x 190	160 x 144	64 x 29
**Slice thickness [mm]** **acq/rec**	10	-	5	5	6	5	1 / 0.5	16 / 8	3.5
**Slice gap**	0		1	1	2	1	-	-	-
**NSA / NEX**	1	3	2	3	2	1	1	1	1
**Bandwidth [Hz/pixel]**	186.9	2540.7	109.3	224.0	152.9	1834	108.8	541.1	60.3

**Table 2:** MRI sequences for lumbar spine scan

	**Sequences for Philips MRI Scanner**					
	**Body Region: Lumbar Spine, Patient Position: Supine, Patient Entry: Head First, Landmark on Trochanter**					
	**Localizer**	**Sagittal T1**	**Sagittal T2**	**Axial T1**	**Axial T2**	**STIR**
	**Landmark on Trochanter**	**Landmark on Trochanter**	**Landmark on Trochanter**	**Landmark on Trochanter**	**Landmark on Trochanter**	**Landmark on Trochanter**
**Name Anatomy Philips**	Spine-lumbar	Spine-lumbar	Spine-lumbar	Spine-lumbar	Spine-lumbar	Spine-lumbar
**Sequence Name Philips**	survey	T1W_TSE_SAG	T2W_TSE_SAG	T1W_TSE(2)_TRA	T2W_TSE(2)_TRA	STIR_longTE_SAG
**plane**	ax/cor/sag	sag	sag	ax	ax	sag
**Scan time [min]**	0:28	02:32	02:12	02:59	03:23	03:13
**stacks**	3	1	1	3	3	1
**slices**	3 each	9	9	5	5	9
**Scan technique**	FFE	SE	SE	SE	SE	IR
**Fast imaging mode**	none	TSE	TSE	TSE	TSE	TSE
**(EPI/Turbo) Factor**		5	21	4	18	17
**TE [ms]**	3.9	8	120	8	120	80
**TR [ms]**	23	380	2825	325	3500	3400
**TI [ms]**	-	-	-	-	-	165
**Flip angle [deg]**	45	90	90	90	90	-
**FOV [mm]**	400	160	160	200	200	160
**Scan [%]**	74.6	72.3	72,7	77.7	76	70.5
**Scan matrix**	268 x 200	176 x 240	176 x 241	224 x 174	224 x 171	176 x 238
**Slice thickness**	10	4	4	4	4	4
**Slice gap**	user defined	default	default	default	default	user defined
**NSA / NEX**	2	4	4	6	6	4
**Bandwidth [Hz/pixel]**	285.9	196.6	324,7	198.4	139.9	297.1

There were certain restrictions to be considered for MR scans: since Evia pacemakers have an exclusion zone for the isocenter landmark (exclusion zone: below the level of the eyes and above the level of the major trochanters), the iso-center landmark had to be placed at the level of the eyes for brain scan, and at the level of the major trochanters for lumbar spine scan, respectively. The patient had to be in a supine position. No additional local sending inductors were allowed. The gradient slew rate had to be ≤ 200 T/m/s. The whole body specific absorption rate (SAR) had to be ≤ 2 W/kg, whereas the head SAR had to be ≤ 3.2 W/kg, respectively. The scanning time per MR session should not exceed 30 minutes.

During MR patients were monitored using a MR compatible telemetry based ECG and pulse oximetry (In vivo Corporation Orlando, FL, USA). ECG and pulse oximetry curves were continuously observed by the first author and recorded on digital video tape for the purpose of (primarily not intended) retrospective evaluation. Additionally patients were able to trigger manually an alarm in case of emergency. Emergency equipment was available onsite. After MR patients were asked for unusual perceptions (e.g. local heating, pacemaker vibration etc.) during MR scans.

### Primary study endpoints

#### Primary endpoint #1: MRI and pacing system related serious adverse device effect (SADE) free-rate

While all adverse events had to be recorded throughout the entire study, only the number of possibly pacing system and MRI related SADEs were the basis for endpoint calculation of the SADE rate. A SADE was pacing system related if it resulted from the presence or performance of the pacing system. A SADE was MR related if it occurred due to the interaction of the pacing system with the MR procedure. This was the case if the patient was within the 5 Gauss line of the MR system or if the SADE occurred in the month following the MR procedure. SADE’s due to programming the pacemaker to MR mode were also classified as MR procedure related. Pocket and lead infections were described but not taken into account for the primary endpoint.

The parameter of interest p_SADE_ was the SADE free rate per patient, which was calculated by 100% - (number of SADE divided by the number of patients)* 100%. It was expected, that this SADE free rate will be greater than 90%.

#### Primary endpoint #2: Pacing threshold rise (atrial and ventricular)

The percentage of pacing leads with a pacing threshold rise between pre-MR follow-up (baseline FU) and 1-month follow-up (2^nd^ FU) was investigated. The threshold behavior of the lead was defined as a success if the increase was not larger than or equal to 1.0 Volts. Only measurements of the same polarity (either uni- or bipolar) were taken into account. The proportion (p_PT_) of pacing threshold successes was calculated by dividing the number of leads without a pacing threshold rise as defined above by the total number of all leads.

### Secondary study end-points

#### P-wave sensing attenuation

The P-wave sensing attenuation rate (p_MRI Psensing_) was the percentage of patients who experienced a P-wave sensing amplitude attenuation. P-wave amplitude attenuation was defined as either a P-wave amplitude decrease (between pre-MRI follow-up and 1-month follow-up) exceeding 50% *or* a P-wave amplitude at 1-month follow-up smaller than 1.5 mV. In case that a patient fulfilled both conditions he/she was counted only once. The proportion (p_MRI Psensing_) was calculated by dividing the number of patients with P-wave sensing attenuation by the total number of patients. Only measurements of the same polarity (either uni- or bipolar) were taken into account. Patients with a P-wave sensing amplitude smaller or equal to 1.5 mV at pre-MRI were excluded from the analysis of this endpoint.

For comparison: In the Setrox S Master Study the percentage of patients with a P-wave sensing amplitude lower than 1.5 mV at the 3-month follow-up was 4/109 = 3.7%. In the same study, the percentage of patients with an amplitude decrease exceeding 50% between 1-month and 3-month follow-up was 1/97 = 1.0%. This one patient had also a sensing amplitude below 1.5 mV and therefore was not counted double. Hence a percentage (p_Setrox Psensing_) of 3.7% resulted. For the purpose of this study the difference between the percentages were calculated via: Δ_P-wave sensing_ = p_MRI Psensing_ – p_Setrox Psensing_ and was expected to be zero or less.

#### R-wave sensing attenuation

The R-wave sensing attenuation rate (p_MRI Rsensing_) was the percentage of patients who experienced a R-wave amplitude attenuation. R-wave amplitude attenuation was defined as either a R-wave amplitude decrease (between pre-MRI follow-up and 1-month follow-up) exceeding 50% *or* a R-wave amplitude at 1-month follow-up smaller than 5.0 mV. In case that a patient fulfilled both conditions he/she was counted only once. The proportion (p_MRI Rsensing_) was calculated by dividing the number of patients with R-wave sensing attenuation by the total number of patients. Only measurements of the same polarity (either uni- or bipolar) were taken into account. Patients with a R-wave sensing amplitude smaller or equal to 5.0 mV at pre-MRI were excluded from the analysis of this endpoint.

For comparison: In the Setrox S Master Study the percentage of patients with a R-wave sensing amplitude lower than 5.0 mV at the 3-month follow-up was 3/61 = 4.9% [8]. In the same study, the percentage of patients with a sensing decrease exceeding 50% between 1-month and 3-month follow-up was 0/51 = 0.0%. Therefore the percentage (p_Setrox Rsensing_) resulted to 4.9% [8].

For the purpose of this study the difference between the percentages was calculated via: Δ_R-wave sensing_ = p_MRI Rsensing_ – p_Setrox Rsensing_ and was expected to be zero or less.

#### Follow-up

Pacemakers were interrogated immediately before (baseline FU) and immediately after (1^st^ FU) MR to assess potential changes of lead parameters (right atrial (RA)/right ventricular (RV) sensing [mV], pacing threshold [V/0.4 ms], pacing impedance [Ohm]) as well as of battery status (100% or less). Patients were followed for 3 months with out-patient PM clinic visits at 1 month (2^nd^ FU) and 3 months (3^rd^ FU) after MR. Additionally, patients were remotely monitored using Home Monitoring based on routinely scheduled 30-day or event triggered transmissions.

### Statistical analyses

Continuous variables are expressed as mean ± SD. The effects of MR on sensing, pacing thresholds, and lead impedance were analysed by one-way ANOVA, for paired data followed by post-hoc analysis (Student’s paired t-test). Categorical variables were compared using the chi-square test and the Fisher’s exact test, where appropriate. Box plots display the following descriptive measurements: sample minimum: lower end of the whisker; Q1 (lower quartile): bottom of the box; Q2 (median): line inside the box; Q3 (upper quartile): top of the box; sample maximum: upper end of the whisker; mean: diamond inside the box; outliers: circles). A p-value < 0.05 was considered significant.

## Results

Thirty-one patients were enrolled. One male patient with an Evia DR-T had to be excluded because of an enrollment violation. The patient underwent scheduled surgery for single coronary bypass of the circumflex artery originating from the right coronary artery 4 weeks after the MR. The surgery was indicated before enrollment. Therefore, 30 patients are included in the following data analyses. Table 3 shows the characteristics of the patient cohort. The leading pacemaker indication was intermittent or permanent higher degree AV block (n = 10 [33%]). Half of the patients had been implanted with dual chamber pacemakers. In a minority of patients (n = 6 [20%]) permanent pacemakers were placed in an intra- or sub pectoral pocket, mainly due to thin subcutaneous fatty tissue. The time from pacemaker implantation to enrollment/MR scan was 2.6 ± 1.7 months (median 1.7 months).

**Table 3:** Patient demographics

	**N**	**%**
**Total patients**	30	100
**Female**	12	40
Age (years)	73 ± 12	
**Height (cm)**	169 ± 0.1	
**Weight (kg)**	80 ± 16	
**Body mass index**	28 ± 5	
**Pacemaker indication**		
Higher degree AV block	10	33
Sick sinus syndrome	6	20
Brady-/Tachy-Syndrome	7	23
AF with significant bradycardia	7	23
**AP (DC-PM only;%; [median])**	34 ± 33 [27]	
**VP (%; [median])**	63 ± 31 [63]	
**AF-Burden (DC-PM only;%; [median])**	11 ± 28 [0]	
**Pacing mode during MRI**		
**A00**	1	3
**V00**	3	10
**D00OFF**	5	17
	21	70
**Implanted pacemakers**		
Evia SR-T	15	50
Evia DR-T	15	50
**Implanted leads**		
RA: Safio S 53 cm	15	50
RV: Safio S 60 cm	30	100
**RV lead position**		
apical	3	10
RVOT/septal	27	90
**Implantation site left pectorally**	30	100
**PM pocket subcutaneously**	24	80
**Time from implantation (months)**	2.6 ± 1.7	

### MR scans

Before MR all pacemakers were followed and were programmed according to the protocol. In all patients who were not pacemaker dependent (n = 21 [70%]) the pacing function was deactivated.

Tables 1 and 2 show the scan protocols for head and lower lumbar spine scan, respectively. Table 4 shows the mean values of SAR, scan duration and gradient strength for the different scan sequences. The total scan time of the brain scan was 14.0 minutes, of the lumbar spine scan 15.2 minutes, respectively.

**Table 4:** Measurements during MR scans

**Scan sequence**		**SAR (W/kg)**	**Scan duration (sec.)**	**Gradient strength (% PNS)**
**Head**				
3 plane localizer	Head CNS-Brain	0.3 ± 0	17.6 ± 3.1	16.0 ± 0
Reference Scan	Ref Scan	0.3 ± 0	78.3 ± 13.9	19.5 ± 1.4
AX SE T1	Head CNS-Brain	2.0 ± 0	101.0 ± 0	12.1 ± 1.6
AX FSE T2	Head CNS-Brain	1.6 ± 0	133.0 ± 0	36.8 ± 0.6
T2 Flair	Head CNS-Brain	0.5 ± 0	198.0 ± 0	30.5 ± 0.6
Diffusion	Head CNS-Function	0.2 ± 0	36.0 ± 0	52.7 ± 1.2
3D TOF MT	Head CNS-Angio	1.8 ± 0	169.0 ± 0	35.9 ± 4.7
CE-MRA	Head CNS-CE-Angio	1.9 ± 0.2	56.0 ± 0	14.0 ± 0
Perfusion	Head CNS-Angio	0.2 ± 0.9	51.0 ± 0	51.2 ± 9.7
Total scan time (min)			14.0	
**Lumbar spine**				
Localizer center at L1	Survey	0.3 ± 0	53.0 ± 0	17.0 ± 0
Sagital T1 Isocenter at L1	T1W_TSE_SAG	2.0 ± 0	158.0 ± 0	38.7 ± 1.3
Sagital T2 Isocenter at L1	T2W_TSE_SAG	2.0 ± 0	132.0 ± 0	40.0 ± 0
Axial T1 Isocenter at L1	T1W_TSE (2)_TRA	2.0 ± 0	179.0 ± 0	40.0 ± 0
Axial T2 Isocenter at L1	T2W_TSE (2)_TRA	1.6 ± 0	203.0 ± 0	39.6 ± 2.0
Sagital T1 Isocenter S1	STIR_long TE_SAG	1.9 ± 0	193.0 ± 0	41.0 ± 0
Total scan time (min)			15.2	

In one patient, metallic artifacts surprisingly were recognized during the 1^st^ (survey) sequence of the head scan. MR was immediately stopped. After performing x-ray demonstrating the metallic artifact (very small splinters) to be outside the osseous head and the decision that MR wouldn’t cause any harm to the patient, the survey sequence was started again and the whole protocol was completed. The metallic splinters were unknown even to the patient and might be the result from working with an angle grinder.

None of the patients reported about uncomfortable feelings or other disorders during MR possibly related to the pacemaker.

MR scans were performed in a non-diagnostic manner, but were analyzable concerning rough conspicuousnesses. Analyses of MR scans revealed no important incidental findings.

After completion of all MR sequences pacemakers were interrogated again and re-programmed. Total time from activation to deactivation of the ProMRI feature was 73 ± 17 minutes (median 74).

### Adverse events

#### MRI related adverse events

No MRI related early or late adverse events occurred. Therefore, the 1^st^ primary endpoint was met (p_SADE_ = 100%).

#### Other adverse events

**Figure 1:**
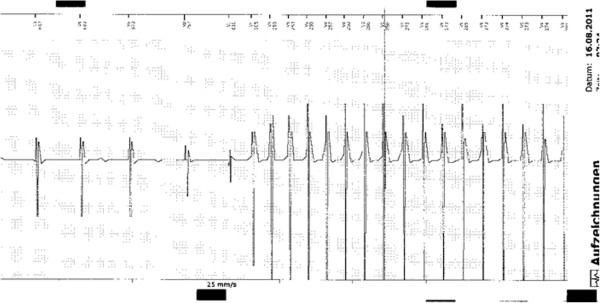
**Patient #31.** Episode print-out of spontaneously occurring VT at the time of myocardial infarction/death.

There were 4 AEs. All AEs were not MR related. One female patient (age 56 years, bradycardia/tachycardia syndrome, normal left ventricular function (LVF) prior to PM implantation, VVI-PM [apical lead position]) suffered twice from cardiac decomposition (1^st^ episode 43 days after MR, 2^nd^ episode 66 days after MR) presumably due to the effects of frequent pacing from the right ventricular apex (ventricular pacing: 61% at baseline FU, 70% at 2^nd^ FU) as well as of intermittent rapidly conducted atrial fibrillation. Echocardiography revealed a significantly reduced LVF at the time of first cardiac decomposition. Another female patient (age 83 years, higher degree AV block, DR-PM) was admitted 11 days after MR to our emergency department due to suspected deep vein thrombosis of the left lower leg (which was excluded). One male patient (age 85 years, coronary artery disease, status post CABG, persistent atrial fibrillation, VVI-PM) died suddenly 17 days after MR. The patient was admitted to our emergency department 10 days after the MR scan because of newly recognized icterus. During the in-hospital stay pancreatic carcinoma with disseminations was diagnosed. On 14^th^ of August, 2 hours after discussion about prognosis of the malignant disease and potential further diagnostic and therapeutic options the patient died suddenly. Autopsy revealed a large myocardial infarction as underlying cause of death. Post mortem PM interrogation showed fast VT at the time of death (Figure 1).

#### Lead measurements

Since one patient died before passing the 1-month FU, calculation of the study endpoints is based on the data of 29 patients. The 1-month follow-up took place after a mean of 1.1 months after the MR procedure, the 3-month follow-up after 3.0 months, respectively.

All lead measurements were done in a bipolar configuration. Except for RV pacing impedance assessed immediately after the MR paired Student’s t-test revealed no significant differences of lead dependent parameters as well as of battery status between the pre-MRI FU and all other FUs. Table 5 gives an overview of all measurements from all available FUs.

**Table 5:** Lead Measurements and Battery status

	**Follow-up**				
**Parameter**	**1**^**st**^**FU (n = 30)**	**2**^**nd**^**FU (n = 30)**	**3**^**rd**^**FU (n = 29)**	**4**^**th**^**FU (n = 29)**	p = *
Time from MRI (months)	-	-	1.1 ± 0.3	3.0 ± 0.3	
RA sensing (mV)	3.2 ± 2.1	3.2 ± 2.3	3.2 ± 2.1	3.1 ± 2.0	n.s.
RA pacing threshold (V@0,4 ms)	0.68 ± 0.18	0.67 ± 0.16	0.71 ± 0.24	0.73 ± 0.15	n.s.
RA pacing impedance (Ohms)	507 ± 55	500 ± 46	520 ± 50	495 ± 59	n.s.
RV sensing (mV)	15.0 ± 6.0	15.0 ± 6.0	14.9 ± 6.5	14.7 ± 6.3	n.s.
RV pacing threshold (V@0,4 ms)	0.78 ± 0.22	0.79 ± 0.20	0.78 ± 0.22	0.82 ± 0.24	n.s.
RV pacing impedance (Ohms)	608 ± 54	599 ± 52^+^	607 ± 47	597 ± 57	n.s.
Battery status (%)	100 ± 0	100 ± 0	100 ± 0	99.7 ± 1.3	n.s.
**Value changes (% [median]; compared with pre MRI FU)**					
RA sensing		−2 ± 20 [0]	+6 ± 43 [0]	+15 ± 57 [8]	
RA PTH		+2 ± 23 [0]	+2 ± 30 [0]	+9 ± 7 [0]	
RA Pimp		−1 ± 4 [0]	+3 ± 6 [4]	−2 ± 11 [0]	
RV sensing		−1 ± 6 [−1]	−2 ± 12 [−2]	−4 ± 14 [−1]	
RV PTH		+2 ± 8 [0]	0 ±12 [0]	7 ± 24 [0]	
RV PImp		−2 ± 2 [−2]	0 ± 6 [0]	−2 ± 7 [0]	
Battery status		0 ± 0 [0]	0 ± 0 [0]	0 ± 1 [0]	

Grey columns: relevant FUs for endpoint calculation.

*Oneway ANOVA.

+p < 0.05 when comparing with pre MRI values (paired t-test).

#### Atrial pacing threshold

**Figure 2:**
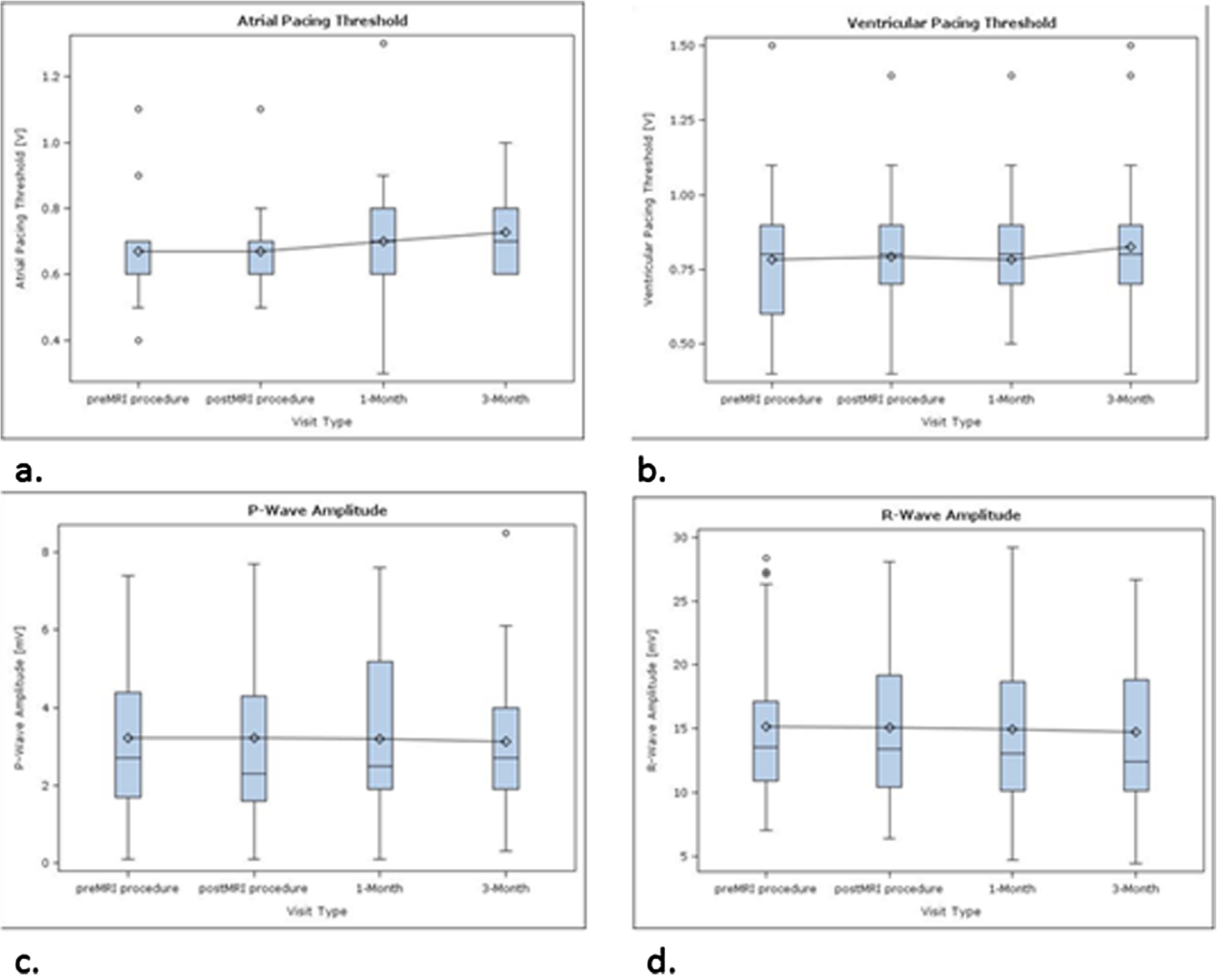
The diagrams show the changes of RA-PTH (a), RV-PTH (b), P wave sensing amplitude (c) and R-wave sensing amplitude (d) at the different FUs.

Atrial pacing thresholds showed a slight increase in the course after the MRI scans (Figure 2a). The measured values were not significantly different between the follow-ups (Table 5). One patient had an increase of the atrial pacing threshold by 75% (from 0.4 V/0.4 ms to 0.7 V/0.4 ms) from the 1^st^ FU to the 2^nd^ FU. There was no further change observed until the 1-month FU.

#### Ventricular pacing threshold

Ventricular pacing thresholds showed a slight increase between the 1-month FU and the 3-month FU (Figure 2b), but the measured differences were not significantly different (Table 5).

The female patient which decompensated twice showed an increase of the ventricular PTH from 0.8 V/0.4 ms at the 1-month FU to 1.5 V/0.4 ms at the 3-month FU (+88%) most likely due to new rhythm control therapy with amiodarone.

All other atrial and ventricular pacing threshold changes observed were less than 50%. None of the patients had an increase of the atrial or ventricular PTH by 1 Volt or more between the baseline FU and the 1-month FU. Therefore, the second primary endpoint was met (p_PT_ = 1).

#### Atrial sensing

Values for atrial sensing were stable throughout the study period (Table 5, Figure 2c). Three patients had an atrial sensing of ≤ 1.5 mV at the pre-MRI FU and – therefore – were excluded from calculation of the first secondary endpoint. Since no one of the remaining patients (n = 12) had an atrial sensing of < 1.5 mV or had a sensing attenuation by ≥50%, also the first secondary endpoint was met (p_MRI Psensing_ = 0.0%). Δ_P-wave sensing_ was calculated to be −3.7%.

#### Ventricular sensing

Values for ventricular sensing were stable throughout the study period (Table 5, Figure 2d). Two patients had no intrinsic R wave (one at the pre-MRI FU, one at the 1-month FU), one patient died before the 1-month-FU. These 3 Patients were excluded from calculation of the second secondary endpoint. Since no one of the remaining patients (n = 27) had a ventricular sensing of < 5.0 mV nor had a sensing attenuation by ≥50%, also the 2^nd^ secondary endpoint was met (p_MRI Rsensing_ = 0.0%). Δ_P-wave sensing_ was calculated to be −6.9%.

#### Home monitoring

Within the observational period more than 1800 data sets were transmitted, composed of - amongst other parameters – RA/RV Impedance, RA/RV Threshold, RA/RV Sample Amplitude, RA/RV Sample Amplitude Mean, Heart Rate (24 h) and Battery Status. Neither hardware related annotations (e.g. pacing impedance alert, battery depletion) were transmitted nor device related (hardware/software) irregularities were found in the status information based on routinely scheduled 30-day or event triggered transmissions.

#### Pacemaker associated MR imaging artifacts

**Figure 3:**
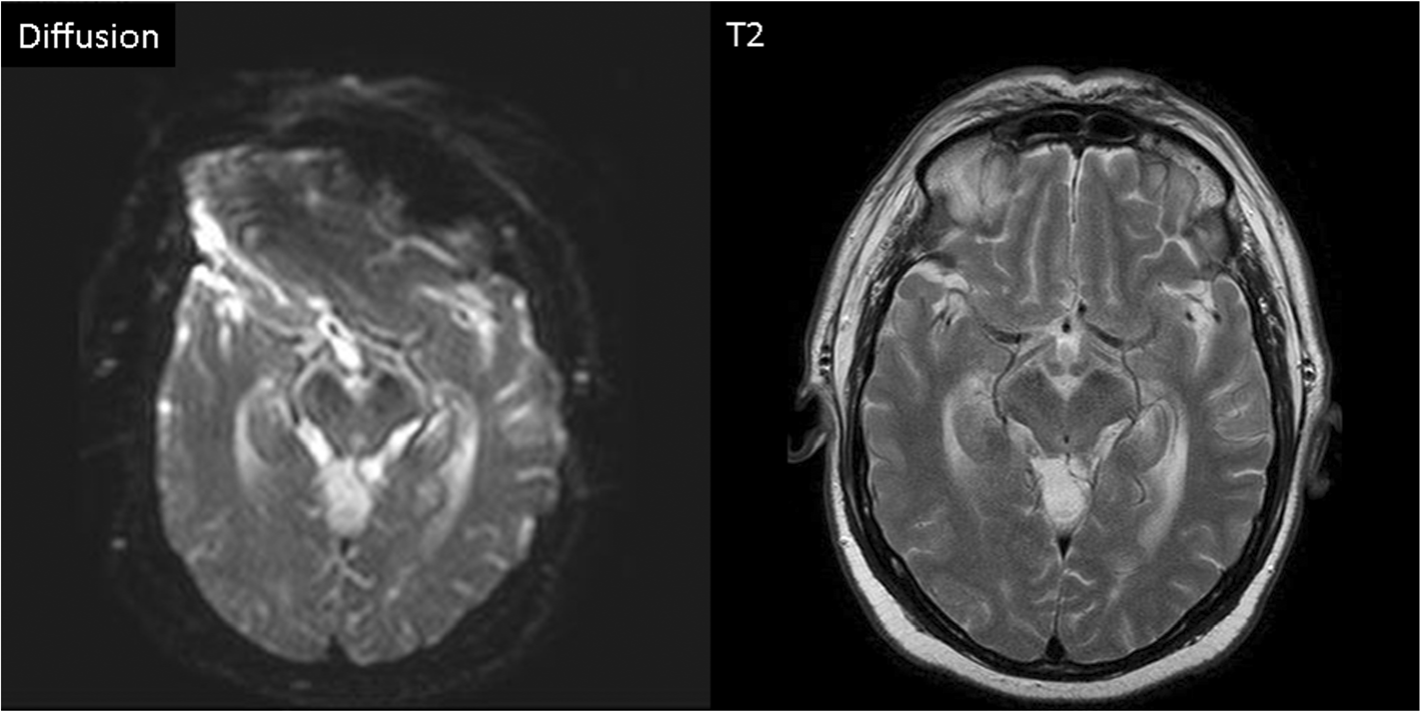
**Left: Diffusion weighted gradient echo image.** Notice pacemaker associated distortion of the image in the frontal region of the brain. **Right: T2** weighted spin echo image (identical slice location) showing normal anatomy not affected by the presence of a pacemaker.

In all patients the presence of the pacemaker caused an inhomogeneity of the static magnetic field in the mid face and the frontal lobe areas resulting in a MR imaging artifact on diffusion weighted sequences of the brain (Figure 3).

## Discussion

This study is the first to report about functionality of the MR conditional Evia pacemaker in a MR environment, and the second study that reports about MR eligible pacemaker systems undergoing MR [8].

The MR conditional Evia pacemakers with Safio S leads demonstrated unobtrusive function under MR conditions. The use of the Evia pacemakers with Safio S leads in MR environment was feasible.

Since there were no MR related SADEs nor were significant pacing threshold increases or sensing attenuations as defined by the protocol all primary and secondary study endpoints were met. Lead measurements were not affected by MR immediately nor showed significant changes within the observational period of 3 months.

The continuous observation of the ECG and pulseoxy monitor showed no inhibition of pacemaker stimulation, sustained or non-sustained atrial and/or ventricular arrhythmias, asystole, or other unexpected changes of heart rate occurred in the context of MR scans. Also no conspicuousnesses concerning pacemaker statistics and the functions or the electrical integrity of the devices occurred during or after MR. No patient reported unusual feelings/sensations that potentially were related to the pacemaker while being in the MR environment. Observed differences in lead measurements between the different follow-ups were in clinically accepted ranges.

The observed pacemaker associated MR imaging artifact of the brain on diffusion weighted images (Figure 3) may have no relevant clinical importance, since the image quality of the remaining sequences such as FLAIR and T2 weighted spin-echo images is not affected (Figure 3).

As recommended patient surveillance during the MRI scans was realized by using a combination of ECG and pulse oximetry [2-7]. Verbal contact with the patient was nearly impossible especially during the brain scan. ECG is sometimes highly affected by radio frequencies during MR scans and only useable in-between the MR sequences. Our experience is that only pulse oximetry in conjunction with a patient activated alert system seems to be reliable for patient surveillance during total scan time, whereas ECG with appropriate quality is reliable only occasionally during the scans.

Another finding of the study is that patients were programmed to the ProMRI mode for a mean of 73 ± 17 minutes. This fact has impact on patient surveillance not only during MR scan, but also before and thereafter. This long period observed in our study was a result from cumulative 30 min MR scans, changing the body position in relation to the MR bore between the two different scans, taking off clothes before and on thereafter, and placement of the technical equipment for the purpose of patient surveillance. Additional time was needed for awaiting availability of the MR scanner since study scans were performed within daily routine of the private radiology institute. Although the pacing function was set in all non-pacemaker dependent patients (n = 21 [70%]) to OFF when we programmed the pacemakers into the ProMRI mode, the long period our patients remained in the ProMRI mode had no impact on patient safety within our study since pacemaker programming was done in or near the control room of the MR scanner, and patients never were on their own. But what to do in the case pacemaker programming is located far away from the MR scanner (e.g. out-patient pacemaker office and radiology department located in different facilities). In this case, programming the pacemaker off may provoke complications like syncope on the way to or back from the scanner. This scenario may be unlikely in this low-risk patient group, but would have legal implications if it happens. There are several ways to overcome this problem. The personnel intensive way is to guide the patient e.g. from the pacemaker out-patient clinic to the MR scanner and later back again. Another way is to remotely activate/deactivate the specific MR mode just when the patient enters/leaves the room where the MR scanner is located. Of course, one could abstain from programming a different than an asynchronous pacing mode. Indeed asynchronous pacing was judged to be safe, but only for a short time as it usually is used during pacemaker follow-up [14]. Seen from this point of view there was (and would have been) long term asynchronous pacing in our cases. This fact may be another source of hidden danger when performing MR even in patients with MR conditional pacemakers and all those who are involved should be aware of this [15].

### Limitations

The major limitation of our study is the limited number of patients and the non-randomized and – therefore – statistically not powered study design. However, the intention of the study was to collect experience in order to prepare the ProMRI AFFIRM master study of the MRI compatibility of the Evia/Entovis pacemaker in combination with Safio S leads.

## Conclusion

The new MR conditional Evia pacemaker system demonstrated unobtrusive function under MR conditions. Observed differences in lead measurements between the different follow-ups were in clinically accepted ranges. No MR related adverse events occurred. Pacemaker related MR imaging artifacts occurred on diffusion weighted sequences of the brain, but may have no clinical relevance.

Our study demonstrated feasibility and safety of Evia single chamber and dual chamber pacemakers with Safio S leads in a MRI environment under well defined conditions. The long duration patients remained in the specific MR pacemaker programming mode (ProMRI) may have impact on patient safety and, therefore – will influence working algorithms for safe performance of MR in patients even with MR conditional pacemaker systems.

## Abbreviations

AE: Adverse event; CABG: Coronar artery bypass graft(ing); CE: European conformity; DR: Dual chamber rate responsive; ECG: Electrocardiogram; Fr: French; FU: Follow-up; IEGM: Intracardiac electrogram; Kg: Kilogramms; MR: Magnetic resonance; MRI: Magnetic resonance imaging; Ms: Millisecond(s); mV: Millivolt(s); PM: Pacemaker(s); PTH: Pacing threshold; RF: Radio frequency; RA: Right atrial; RV: Right ventricular; RVOT: Right ventricular outflow tract; SADE: System related serious adverse device effect; SAR: Specific absorption rate; SD: Standard deviation; SR: Single chamber rate responsive; V: Volt(s); VP: Ventricular pacing; VT: Ventricular tachycardia; W: Watt(s).

## Competing interests

^1^ Biotronik (lecture fees, travel grants), Boston Scientific (lecture fees, travel grants), Medtronic (travel grants), St. Jude Medical (lecture fees, travel grants).

^2^ None.

^3^ Biotronik (lecture fees, travel grants), Boston Scientific (lecture fees, travel grants), Medtronic (lecture fees, travel grants), St. Jude Medical (lecture fees, travel grants).

^4^ Biotronik employee.

^5^ Medtronic (lecture fees, travel grants).

## Authors’ contributions

CGW substantial contributions to conception and design; acquisition of data, and analysis and interpretation of data; drafting the manuscript; final approval of the version to be published. ES substantial contributions to conception and design; interpretation of data; drafting the manuscript; final approval of the version to be published. PV revising it critically for important intellectual content; final approval of the version to be published. BN substantial contributions to conception and design; acquisition of data, and analysis and interpretation of data; revising manuscript critically for important intellectual content; final approval of the version to be published. HM substantial contributions to conception and design; revising manuscript critically for important intellectual content; final approval of the version to be published. All authors read and approved the final manuscript.
